# Application of IMU/GPS Integrated Navigation System Based on Adaptive Unscented Kalman Filter Algorithm in 3D Positioning of Forest Rescue Personnel

**DOI:** 10.3390/s24185873

**Published:** 2024-09-10

**Authors:** Shengli Pang, Bohan Zhang, Jintian Lu, Ruoyu Pan, Honggang Wang, Zhe Wang, Shiji Xu

**Affiliations:** College of Communication and Information Engineering, Xi’an University of Posts and Telecommunications, Xi’an 710121, China; pangshengli@xupt.edu.cn (S.P.); lujintian@stu.xupt.edu.cn (J.L.); panruoyu@xupt.edu.cn (R.P.); wanghonggang@xupt.edu.cn (H.W.); wangzhe1201@stu.xupt.edu.cn (Z.W.); xushiji@stu.xupt.edu.cn (S.X.)

**Keywords:** global positioning system, inertial measurement unit, adaptive filtering, unscented Kalman filter, emergency rescue, three-dimensional positioning

## Abstract

Utilizing reliable and accurate positioning and navigation systems is crucial for saving the lives of rescue personnel and accelerating rescue operations. However, Global Navigation Satellite Systems (GNSSs), such as GPS, may not provide stable signals in dense forests. Therefore, integrating multiple sensors like GPS and Inertial Measurement Units (IMUs) becomes essential to enhance the availability and accuracy of positioning systems. To accurately estimate rescuers’ positions, this paper employs the Adaptive Unscented Kalman Filter (AUKF) algorithm with measurement noise variance matrix adaptation, integrating IMU and GPS data alongside barometric altitude measurements for precise three-dimensional positioning in complex environments. The AUKF enhances estimation robustness through the adaptive adjustment of the measurement noise variance matrix, particularly excelling when GPS signals are interrupted. This study conducted tests on two-dimensional and three-dimensional road scenarios in forest environments, confirming that the AUKF-algorithm-based integrated navigation system outperforms the traditional Extended Kalman Filter (EKF), Unscented Kalman Filter (UKF), and Adaptive Extended Kalman Filter (AEKF) in emergency rescue applications. The tests further evaluated the system’s navigation performance on rugged roads and during GPS signal interruptions. The results demonstrate that the system achieves higher positioning accuracy on rugged forest roads, notably reducing errors by 18.32% in the north direction, 8.51% in the up direction, and 3.85% in the east direction compared to the EKF. Furthermore, the system exhibits good adaptability during GPS signal interruptions, ensuring continuous and accurate personnel positioning during rescue operations.

## 1. Introduction

In emergency rescue operations, reliable and accurate positioning and tracking systems are crucial for saving lives and expediting rescue efforts. However, in extreme environments such as dense forests, standard GPS often fails to provide stable signals, thereby affecting the efficiency and safety of rescue missions [1,2]. Rescue personnel frequently operate in varied environments, making sole reliance on GPS insufficient for accurate positioning [3,4]. Conventional methods, such as pre-installed beacons or base stations, are impractical due to the unpredictability and suddenness of rescue operations [5,6]. Thus, developing sensor fusion technology that integrates GPS with other sensors is essential for achieving a highly reliable positioning system.

Multi-sensor fusion leverages the unique advantages of different types of sensors to improve estimation accuracy and robustness. Inertial Measurement Units (IMUs) complement GPS systems well [7], enhancing GPS accuracy and mitigating positioning discontinuities caused by GPS signal loss. IMU sensors, being compact, lightweight, and cost-effective, are suitable for embedding in footwear [8] and are widely used in pedestrian inertial navigation [9], employing zero-velocity update (ZUPT) methods to mitigate the speed error drift [10]. Multi-sensor fusion typically employs filters for sensor data integration [11]. The Extended Kalman Filter (EKF) is widely used because it can predict accurate navigation information within a certain range of system nonlinearity when GPS signals are available [12]. However, the EKF has limitations, requiring precise random error models for each sensor [13], which are difficult to model, especially for IMUs [14]. The EKF linearizes nonlinear systems using a Taylor expansion, but this approach often fails in highly nonlinear systems [15]. Consequently, the EKF navigation accuracy significantly degrades when GPS signals are lost, especially in systems with strong IMU nonlinearity.

Rossi et al. utilized the Unscented Kalman Filter (UKF) to fuse data from accelerometers, Global Navigation Satellite System (GNSS) instruments, and rotational motion sensors [16]. Compared to the EKF, the UKF achieves second-order or higher accuracy in estimating the posterior mean and covariance of Gaussian nonlinear system states. However, in practical applications, the UKF uses a fixed noise covariance and lacks adaptive adjustment for measurement and process noise, potentially underperforming with non-standard or unexpected signals. Giseo Park proposed a novel vehicle positioning algorithm based on GPS and IMU sensor fusion, applying the Adaptive Unscented Kalman Filter (AUKF) to combine these complementary sensors for improved position estimation accuracy [17]. However, this algorithm is limited to vehicle motion models and cannot perform three-dimensional positioning [18], restricting its applicability in dynamically adapting to complex environments and achieving precise 3D positioning for rescue personnel.

This study proposes a high-precision positioning technique combining IMUs and GPS, utilizing an Unscented Kalman Filter (UKF) with the adaptive adjustment of the measurement noise covariance matrix for data fusion. The proposed AUKF algorithm enhances the state estimation accuracy, particularly when GPS signals are unstable or interrupted. To further improve the positioning accuracy and practicality, this study also incorporates a barometer to measure the altitude of rescue personnel, achieving precise 3D positioning in complex environments.

Compared to existing positioning methods [19], the proposed AUKF positioning algorithm offers the following advantages: (**1**) This method is based on the standard UKF and includes adjustments to the adaptive covariance matrix of measurement noise, which changes to appropriate values depending on the situation. Specifically, the algorithm quickly increases the magnitude of each element within the covariance matrix when the GPS measurement accuracy sharply declines, effectively reducing the weight of GPS observations. Additionally, by linearizing the nonlinear model, the AUKF can compute the adaptive covariance matrix in real time [20], providing accurate position estimates of rescue personnel considering the nonlinearities of the system and GPS measurement models. (**2**) When a significant drop in GPS measurement signal accuracy is detected, indicating a GPS interruption, the AUKF can promptly adjust the noise covariance matrix values associated with measurement errors. This capability ensures high robustness in the position estimation of rescue personnel, even during GPS outages.

This study conducted tests on two-dimensional-plane and three-dimensional-space road scenarios in forest environments to further evaluate the system’s three-dimensional navigation performance on rugged roads and with GPS signal interruptions. The results demonstrate the superior positioning accuracy of the system in forests compared to the EKF, UKF, and AEKF. Additionally, the IMU employs a real-time-corrected zero-velocity update (ZUPT) algorithm [21], ensuring that the AUKF algorithm effectively mitigates position estimation errors caused by GPS signal loss, thereby ensuring continuous and accurate personnel positioning during rescue operations.

The structure of this paper is as follows: Section 2 describes the design of the wearable device used in this study. Section 3 introduces the basic principles of the UKF and the findings related to the AUKF algorithm. Section 4 presents the experimental test scenarios and results. Concluding remarks are presented in Section 5.

## 2. The Design of the Wearable Device

To develop a reliable and precise positioning and navigation system for rescue personnel, this study combines GPS and IMUs to independently design a wearable device. The hardware design of this device is divided into four modules: the main control module, the sensor module, the communication module, and the power module.

**Figure 1:**
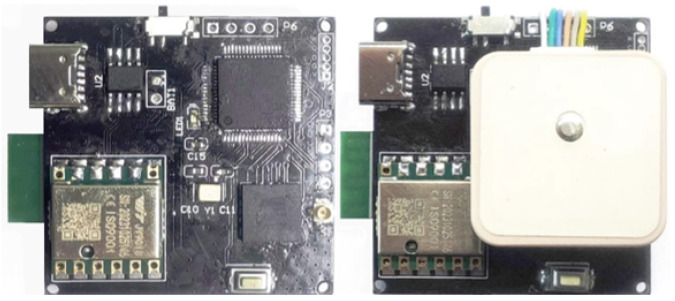
Physical diagram of wearable device hardware.

The main control module utilizes an STM32 microcontroller, which is responsible for parsing and processing data from the IMU and GPS sensors. The sensor module includes a JY-901B sensor and a standalone GPS module. The IMU sensor integrates a high-precision gyroscope, accelerometer, magnetometer, and altimeter, capable of capturing gait and position data. The communication module consists of a wireless local area network (WLAN) that transmits the collected rescue personnel’s walking posture and position data to the host computer. The power module includes a lithium ion battery, a lithium ion battery charging module, and a voltage regulator. The dimensions of the wearable device are 35mm×32mm×12mm, and its hardware layout is shown in Figure 1.

## 3. AUKF Algorithm for Integrated Navigation

### 3.1. The Traditional UKF Algorithm

Considering a nonlinear discrete system, its state and measurement equations are as follows:

(1) $$\left\{\begin{array}{c} X_{k}=f\left(X_{k-1},u_{k},W_{k}\right)\\ Z_{K}=H_{k}X_{k}+V_{k} \end{array}\right.$$

Here, $X_{k}$, $u_{k}$, and $Z_{K}$ represent the system state vector, deterministic input term, and measurement vector at time *k*, respectively [22]; f(•) is a nonlinear function; $H_{k}$ is the measurement matrix; and system noise $W_{k}$ and measurement noise $V_{k}$ are uncorrelated zero-mean Gaussian white noise sequences with the following statistical properties:

(2) $$\left\{\begin{array}{c} E\left[W_{k}\right]=0,\mathrm{cov}\left(W_{k},W_{j}\right)=Q_{k}\delta_{kj}\\ E\left[V_{k}\right]=0,\mathrm{cov}\left(V_{k},V_{j}\right)=R_{k}\delta_{kj}\\ \mathrm{cov}(W_{k},V_{j})=0 \end{array}\right.$$

In Equation (2), $Q_{k}$ is a non-negative definite matrix, and $R_{k}$ is a positive definite matrix, representing the variance matrices of $W_{k}$ and $V_{k}$, respectively. $\delta_{kj}$ is the Kronecker function.

The system state vector and system noise vector are combined into the augmented state vector for the standard UKF algorithm $X^{a}$:

(3) $$\begin{array}{c} X^{a}={\left[\begin{array}{cc} X & W \end{array}\right]}^{T}\\ \chi^{a}={\left[\begin{array}{cc} {\left(\chi^{X}\right)}^{T} & {\left(\chi^{W}\right)}^{T} \end{array}\right]}^{T} \end{array}$$

Here, $\chi^{a}$, $\chi^{X}$, and $\chi^{W}$ are the sample point vectors of $X^{a}$, $X_{k}$, and $W_{k}$, with dimensions *n*, $L_{x}$, and $L_{w}$, respectively, where $n=L_{x}+L_{w}$.

The basic process of the UKF algorithm is as follows:

Step (1). Initialization

(4) $${\hat{X}}_{0}=E\left[X_{0}\right]$$

(5) $$P_{0}=E\left[\left(X_{0}-{\hat{X}}_{0}\right){\left(X_{0}-{\hat{X}}_{0}\right)}^{T}\right]$$

(6) $${\hat{X}}_{a_{0}}=E\left[X^{a}\right]={\left[\begin{array}{cc} {\hat{X}}_{0}^{T} & 0 \end{array}\right]}^{T}$$

(7) $$P_{0}^{a}=E\left[X^{a}\right]=E\left[\left(X_{0}^{a}-{\hat{X}}_{0}^{a}\right){\left(X_{0}^{a}-{\hat{X}}_{0}^{a}\right)}^{T}\right]=\left[\begin{array}{cc} P & 0\\ 0 & Q \end{array}\right]$$

Step (2). Sample Point Calculation

(8) $$\chi_{i,k-1}^{a}={\hat{X}}_{k-1}^{a},i=0$$

(9) $$\chi_{i,k-1}^{a}={\hat{X}}_{k-1}^{a}+{\left(\sqrt{\left(n+\lambda \right)P_{k-1}^{a}}\right)}_{i},i=1,2,\cdots ,n$$

(10) $$\chi_{i,k-1}^{a}={\hat{X}}_{k-1}^{a}-\left(\sqrt{\left(n+\lambda \right)P_{k-1}^{a}}\right),i=n+1,n+2,\cdots ,2n$$

Here, ${\hat{X}}_{k-1}^{a}$ and $P_{k-1}^{a}$ are the state estimate and error covariance at time k−1;

${\left(\sqrt{\left(n+\lambda \right)P_{k-1}^{a}}\right)}_{i}$ is the *i*-th column of the square root (Cholesky decomposition) matrix [23] of $\sqrt{\left(n+\lambda \right)P_{k-1}^{a}}$.

(11) $$W_{i}^{m}=\frac{\lambda }{n+\lambda },i=0$$

(12) $$W_{i}^{c}=\frac{\lambda }{n+\lambda }+\left(1-\alpha^{2}+\beta \right),i=0$$

(13) $$W_{i}^{m}=W_{i}^{c}=\frac{1}{2(n+\lambda )},i=1,2,\cdots ,2n$$

Here, $W_{i}^{m}$ and $W_{i}^{c}$ are the mean and covariance weights, respectively; $\lambda =\alpha^{2}\left(n+\kappa \right)-n$ is the correction factor; and the constant λ determines the distribution of sigma points around ${\hat{X}}_{k-1}^{a}$. Parameters α, β, and κ are scaling factors, where α and κ can be used to match the higher moments of the random variable [24].

Step (3). Time Update

(14) $$\chi_{i,k|k-1}^{X}=f\left(\chi_{i,k-1}^{X},\chi_{i,k-1}^{W}\right),i=0,1,\cdots ,2n$$

(15) $${\hat{X}}_{k|k-1}=\sum_{i=0}^{2n}W_{i}^{m}\chi_{i,k|k-1}^{X}$$

(16) $$P_{k|k-1}=\sum_{i=0}^{2n}W_{i}^{c}\left[\chi_{i,k|k-1}^{X}-{\hat{X}}_{k|k-1}\right]{\left[\chi_{i,k|k-1}^{X}-{\hat{X}}_{k|k-1}\right]}^{T}+Q_{k}$$

Step (4). Measurement Update

(17) $${\hat{Z}}_{k|k-1}=H_{k}{\hat{X}}_{k|k-1}$$

(18) $$P_{{\hat{Z}}_{k|k-1}}=H_{k}P_{k|k-1}H_{k}^{T}+R_{k}$$

(19) $$P_{{\hat{X}}_{k|k-1}{\hat{Z}}_{k|k-1}}=P_{k|k-1}H_{k}^{T}$$

Step (5). Filtering Update

(20) $$K_{k}=P_{{\hat{X}}_{k|k-1}{\hat{Z}}_{k|k-1}}P_{{\hat{Z}}_{k|k-1}}^{-1}$$

(21) $${\hat{X}}_{k}={\hat{X}}_{k|k-1}+K_{k}\left(Z_{k}-{\hat{Z}}_{k|k-1}\right)$$

(22) $$P_{k}=P_{k|k-1}-K_{k}P_{{\hat{Z}}_{k|k-1}}K_{k}^{T}$$

### 3.2. AUKF Design

The standard UKF algorithm performs well in handling nonlinear problems. However, when the statistical characteristics of measurement noise are unknown or inaccurate, it may lead to decreased filtering accuracy or even divergence [25]. Equation (21) shows that if measurement data $Z_{k}$ contain outliers and the system continues to use the initialized measurement noise covariance matrix $R_{k}$ without timely adjustment, it fails to suppress the impact of outlier measurements on filtering, resulting in the unclear convergence or even divergence of the filter. Therefore, the adaptive adjustment of the system noise covariance matrices $Q_{k}$ and measurement noise covariance matrices $R_{k}$ during filtering would significantly enhance the estimation characteristics of the Kalman filter and its ability to suppress outliers [26].

However, in practical applications, simultaneously adapting $Q_{k}$ and $R_{k}$, requiring the Cholesky decomposition of $P_{k-1}^{a}$, which can be unstable for IMU and GPS combined navigation, especially when the IMU is used for state prediction, can lead to instability. Therefore, this paper proposes an Adaptive Unscented Kalman Filter method for measurement noise covariance matrices $R_{k}$. The system state and measurement equations are given by Equation (1), in contrast to Equation (1), where $R_{k}$ is a known Gaussian white noise covariance matrix; here, $R_{k}$ is an unknown quantity. This paper adopts the Sage-Husa adaptive filtering approach to estimate the measurement noise at the current time, with the prediction error (innovation) formula as follows:

(23) $${\tilde{Z}}_{k|k-1}=Z_{k}-{\hat{Z}}_{k|k-1}=H_{k}X_{k}+V_{k}-H_{k}{\hat{X}}_{k|k-1}=H_{k}{\tilde{X}}_{k|k-1}+V_{k}$$

Under the assumption of unbiased initial state selection, the further prediction error ${\tilde{X}}_{k|k-1}$ remains unbiased. Given that the mean of the measurement noise $V_{k}$ is zero, it follows that the mean of the innovation ${\tilde{Z}}_{k|k-1}$ is also zero. Considering the mutual independence between ${\tilde{X}}_{k|k-1}$ and $V_{k}$, the variance of the measurement noise covariance matrix can be derived by simultaneously taking the variance of Equation (23) and rearranging:

(24) $$R_{k}=E\left[{\tilde{Z}}_{k|k-1}{\tilde{Z}}_{k|k-1}^{T}\right]-H_{k}P_{k|k-1}H_{k}^{T}$$

Here, $E[{\tilde{Z}}_{k|k-1}{\tilde{Z}}_{k|k-1}^{T}]$ theoretically represents the ensemble average of a random sequence. However, in practical applications of adaptive filtering algorithms, it is replaced by a time average. The recursive estimation method with equal weighting for $R_{k}$ can be constructed as follows:

(25) $${\hat{R}}_{k}=\left(1-\frac{1}{k}\right){\hat{R}}_{k-1}+\frac{1}{k}\left({\tilde{Z}}_{k|k-1}{\tilde{Z}}_{k|k-1}^{T}-H_{k}P_{k|k-1}H_{k}^{T}\right)$$

Here, the initial value ${\hat{R}}_{0}$ can be chosen based on practical considerations to be sufficiently large in variance.

In Equation (25), when k→∞, it yields 11kk→0, indicating that the adaptive capability gradually diminishes after long-term filtering, nearly losing its adaptive effectiveness. To consistently maintain the appropriate adaptive capability, the equal weighting in Equation (25) can be modified to an exponentially decaying memory-weighted average as follows:

(26) $${\hat{R}}_{k}=\left(1-\beta_{k}\right){\hat{R}}_{k-1}+\beta_{k}\left({\tilde{Z}}_{k|k-1}{\tilde{Z}}_{k|k-1}^{T}-H_{k}P_{k|k-1}H_{k}^{T}\right)$$

(27) $$\beta_{k}=\frac{\beta_{k-1}}{\beta_{k-1}+b}$$

Here, $\beta_{0}=1$ denotes the initial value, and 0<b<1 represents the decaying factor. As *k* becomes sufficiently large, $\beta_{k}\approx 1-b$, where a smaller *b* enhances the adaptive capability to variations in new measurement noise. In this study, b=0.995.

Assuming that ${\hat{R}}_{k}$ is a diagonal matrix, sequential filtering updates the *i*-th scalar measurement sequentially with the scalar measurement equation:

(28) $$\rho_{k}^{\left(i\right)}={\left({\tilde{Z}}_{k|k-1}^{\left(i\right)}\right)}^{2}-H_{k}^{\left(i\right)}P_{k|k-1}^{\left(i\right)}{\left(H_{k}^{\left(i\right)}\right)}^{T}$$

Additionally, the lower limit condition $R_{\min}^{\left(i\right)}$ ensures that $R_{k}^{\left(i\right)}$ remains positive, while the upper limit condition $R_{\max}^{\left(i\right)}$ quickly reduces the reliability of measurement $Z_{k}^{\left(i\right)}$. $R_{\max}^{\left(i\right)}$ is also used to detect measurement anomalies, in which case the current measurement update is discarded.

(29) $${\hat{R}}_{k}^{\left(i\right)}=\left\{\begin{array}{cc} \left(1-\beta_{k}\right){\hat{R}}_{k-1}^{\left(i\right)}+\beta_{k}R_{\min}^{\left(i\right)} & \mathrm{if}\;0\leq \rho_{k}^{\left(i\right)}\leq R_{\min}^{\left(i\right)}\\ R_{\max}^{\left(i\right)} & \mathrm{if}\;\rho_{k}^{\left(i\right)}>R_{\max}^{\left(i\right)}\\ \left(1-\beta_{k}\right){\hat{R}}_{k-1}^{\left(i\right)} & \mathrm{if}\;\rho_{k}^{\left(i\right)}<0 \end{array}\right.$$

When $\rho_{k}^{\left(i\right)}<0$, sacrificing some accuracy ensures the positive definiteness of *R* to maintain the stability of the filtering algorithm. Setting $\rho_{k}^{\left(i\right)}=0$ eliminates subtractive terms in Equation (29), ensuring that ${\hat{R}}_{k}^{\left(i\right)}$ remains positive definite. If $\rho_{k}^{\left(i\right)}>R_{\max}^{\left(i\right)}$, indicating the imminent divergence of the filter, the current measurement noise *R* is deemed unreliable, suggesting system failure, possibly due to measurement anomalies, prompting the abandonment of the current measurement update.

Employing a sequential adaptive filtering algorithm [27], this approach consistently confines the measurement noise $R_{k}^{\left(i\right)}$ within the interval $\left[R_{\min}^{\left(i\right)},R_{\max}^{\left(i\right)}\right]$, thereby enhancing the adaptive capability and filtering reliability.

### 3.3. IMU/GPS Integrated Navigation System Model Design

The navigation coordinate system (*n* system) is defined in the geographic coordinate frame, i.e., the “east, north, up” (ENU) system. The IMU body frame is referred to as the *b* system, and the computed platform frame is the *p* system. The Earth-centered inertial frame is referred to as the *i* system, and the Earth frame is called the *e* system. The state equation propagates significant attitude errors, with the difference between IMU and GPS outputs used as measurements.

#### 3.3.1. System State Equation

The 15-dimensional state vector $X_{k}=\left[\begin{array}{ccccccccccccccc} \delta \phi_{x} & \delta \phi_{y} & \delta \phi_{z} & \delta v_{x}^{n} & \delta v_{y}^{n} & \delta v_{z}^{n} & \delta p_{x}^{n} & \delta p_{y}^{n} & \delta p_{z}^{n} & \varepsilon_{x}^{n} & \varepsilon_{y}^{n} & \varepsilon_{z}^{n} & \nabla_{x}^{n} & \nabla_{y}^{n} & \nabla_{z}^{n} \end{array}\right]$ is defined with $\delta \phi_{x}$, $\delta \phi_{y}$, and $\delta \phi_{z}$ representing the three-axis attitude angle errors at time *k*. $\delta v_{x}^{n}$, $\delta v_{y}^{n}$, and $\delta v_{z}^{n}$ denote velocity errors in the east, north, and up directions, while $\delta p_{x}^{n}$, $\delta p_{y}^{n}$, and $\delta p_{z}^{n}$ represent position errors in the same directions. $\varepsilon_{x}^{n}$, $\varepsilon_{y}^{n}$, and $\varepsilon_{z}^{n}$ are gyroscope biases, and $\nabla_{x}^{n}$, $\nabla_{y}^{n}$, and $\nabla_{z}^{n}$ are accelerometer biases.

Due to the different coordinate spaces between the vehicle and the navigation coordinate systems, the misalignment angle $\phi^{n}$ between the *n* system and *p* system is expressed in the navigation coordinate system as

(30) $$\phi ={\left[\begin{array}{ccc} \phi_{x} & \phi_{y} & \phi_{z} \end{array}\right]}^{T}$$

The attitude error model of the IMU is described by

(31) $$\dot{\psi }=C_{\phi }^{-1}\left[\left(I-C_{n}^{p}\right)\omega_{in}^{n}+C_{n}^{p}\delta \omega_{in}^{n}-C_{b}^{p}\delta \omega_{ib}^{b}\right]$$

Here, $\delta \omega_{in}^{n}$ represents gyro calculation errors and $\delta \omega_{ib}^{b}$ gyro measurement errors, with the coefficient matrix $C_{\phi }^{-1}$ expressed as

(32) $$C_{\phi }^{-1}=\frac{1}{\cos\phi_{x}}\left[\begin{array}{ccc} \cos\phi_{x}\cos\phi_{y} & 0 & \cos\phi_{x}\sin\phi_{y}\\ \sin\phi_{x}\sin\phi_{y} & \cos\phi_{y} & -\sin\phi_{x}\sin\phi_{y}\\ -\sin\phi_{y} & 0 & \cos\phi_{y} \end{array}\right]$$

The rotation transformation relationship from the *n* system to the *p* system can be described by the coordinate transformation matrix $C_{n}^{p}$ as

(33) $$C_{n}^{p}=\left[\begin{array}{ccc} \cos\phi_{y}\cos\phi_{z}+\sin\phi_{x}\sin\phi_{y}\sin\phi_{z} & -\cos\phi_{y}\sin\phi_{z}+\sin\phi_{x}\sin\phi_{y}\sin\phi_{z} & -\cos\phi_{x}\sin\phi_{y}\\ \cos\phi_{x}\sin\phi_{z} & \cos\phi_{x}\cos\phi_{z} & \sin\phi_{x}\\ \sin\phi_{y}\sin\phi_{z}-\sin\phi_{x}\sin\phi_{y}\sin\phi_{z} & -\sin\phi_{y}\sin\phi_{z}-\sin\phi_{x}\sin\phi_{y}\sin\phi_{z} & \cos\phi_{x}\cos\phi_{y} \end{array}\right]$$

The velocity error model of the IMU is

(34) $$\delta {\dot{v}}^{n}=\left(C_{n}^{p}-I\right)C_{b}^{n}f^{b}+C_{b}^{p}\delta f^{b}+\left(2\delta \omega_{ie}^{n}+\delta \omega_{en}^{n}\right)\times v^{n}-\left(2\omega_{ie}^{n}+\omega_{en}^{n}\right)\times \delta v^{n}$$

Here, $\delta f^{b}$ represents accelerometer measurement errors, $\delta v^{n}$ denotes velocity calculation errors, and $\delta \omega_{in}^{n}=\delta \omega_{ie}^{n}+\delta \omega_{en}^{n}$.

The error model for the IMU position is described by

(35) $$\delta \dot{L}=\frac{\delta v_{N}^{n}}{R_{M}+h}$$

(36) $$\delta \dot{\lambda }=\frac{\delta v_{E}^{n}}{R_{N}+h}\sec L+\delta L\frac{v_{E}^{n}}{R_{N}+h}\tan L\sec L$$

(37) $$\delta \dot{h}=\delta v_{U}$$

For gyroscope and accelerometer bias terms in the state vector, it is ensured that ${\dot{\varepsilon }}_{bx}={\dot{\varepsilon }}_{by}={\dot{\varepsilon }}_{bz}=0$ and ${\dot{\nabla }}_{bx}={\dot{\nabla }}_{by}={\dot{\nabla }}_{bz}=0$.

#### 3.3.2. System Measurement Equation

GPS-derived position information is used for the periodic filtering and calibration of the IMU. The measurement equation takes the difference between GPS and IMU three-dimensional positions and velocities, defined as follows:

(38) $$Y=\left[\begin{array}{c} P_{IMUx}-P_{GPSx}\\ P_{IMUy}-P_{GPSy}\\ P_{IMUz}-P_{GPSz} \end{array}\right]$$

(39) $$H=\left[\begin{array}{ccc} O_{3\times 6} & I_{3\times 3} & O_{3\times 6} \end{array}\right]$$

In Equation (39), $O_{3\times 6}$ denotes a 3×6 dimensional zero matrix, and $I_{3\times 3}$ represents a 3×3 dimensional identity matrix.

## 4. Experimental Results

**Figure 2:**
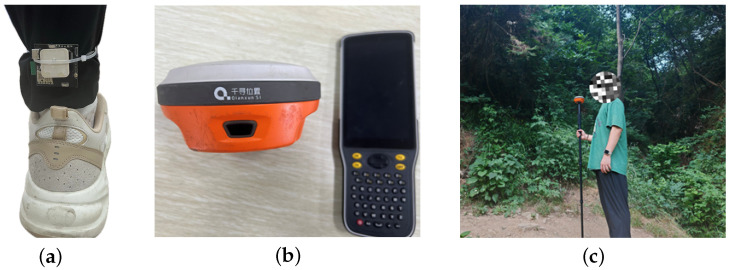
Data collection techniques in rescue operations: (**a**) location of rescue personnel wearable devices; (**b**) high-precision RTK equipment; (**c**) pose when data are collected using a high-precision RTK device.

To enhance the accuracy and reliability of the algorithm for superior positioning results, this study utilized a wearable device worn on the ankles of emergency rescue personnel, as shown in Figure 2a. Rescue personnel walk continuously at speeds of 1.5–2 m/s, with the IMU sampling rate set at 200 Hz and GPS at 10 Hz, to collect posture and positional data during movement.

Two-dimensional plane positioning tests in forests validated the performance of the designed GPS/IMU integrated navigation system in wearable devices for emergency rescue personnel, including its feasibility under conditions causing partial GPS signal loss. Three-dimensional space tests in forests further validated the system’s navigation performance on rugged mountain roads and with a partial GPS signal loss.

Table 1 summarizes the key technical specifications of the sensors used in this study, focusing on the Root-Mean-Square Error (RMS) and circular error probable (CEP) of the IMU and GPS, as well as the positioning accuracy of Real-Time Kinematics (RTK). To evaluate the performance of the Adaptive Unscented Kalman Filter (AUKF) algorithm with measurement noise variance adaptation, this study used high-precision RTK output as the ground truth, comparing the performance of the AUKF in wearable devices with the traditional UKF, EKF, and AEKF algorithms extensively. Figure 2b and Figure 2c illustrate the positioning of high-precision RTK devices and the data collection of the trajectory ground truth using high-precision RTK devices, respectively. The experimental results demonstrate that the AUKF algorithm consistently provides higher positioning accuracy and reliability in various environments, especially delivering more precise positioning accuracy under conditions causing partial GPS signal loss. These findings substantiate the practicality and advantages of the measurement-noise-variance-adaptive AUKF algorithm in complex navigation environments.

### 4.1. Two-Dimensional Plane Positioning Verification

**Figure 3:**
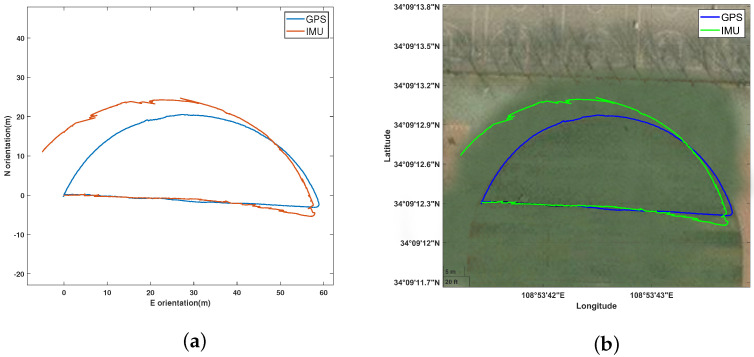
Comparison of 2D planar trajectories between GPS and IMU: (**a**) 2D trajectories; (**b**) actual 2D trajectories.

In the 2D-plane tests conducted in a forest, this study focused on the performance of position estimation and the feasibility of the algorithm when GPS is partially unavailable. As shown in Figure 3, the trajectory comparison using a zero-velocity update algorithm for a single IMU and a single GPS shows that within a 40 m eastward distance, the IMU data have a smaller error. However, beyond 40 m, the IMU error gradually increases, affecting the trajectory accuracy.

**Figure 4:**
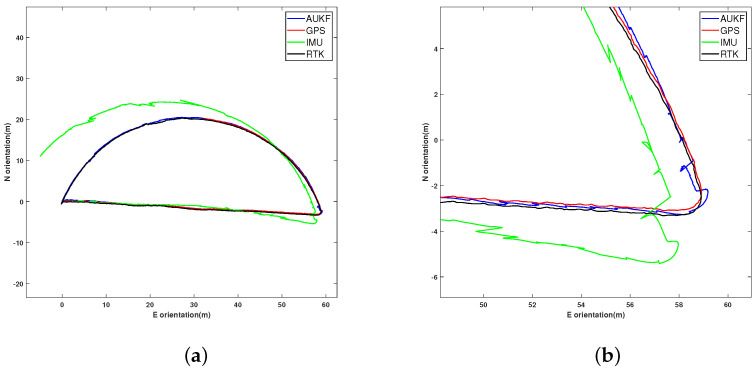
Comparison of AUKF combined navigation, GPS, and IMU errors with ground truth: (**a**) global trajectory; (**b**) detailed local trajectory.

**Figure 5:**
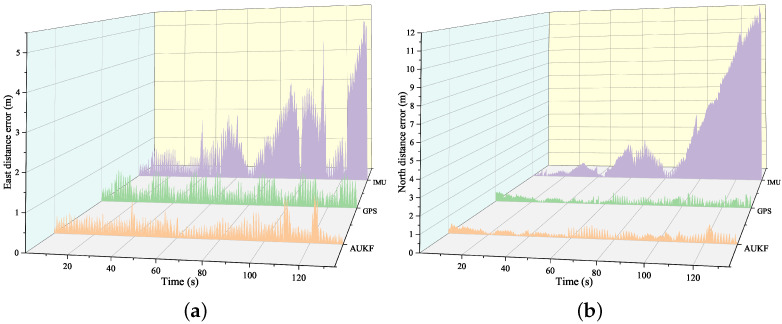
Comparison of AUKF combined navigation, GPS, and IMU errors with ground truth: (**a**) east distance error; (**b**) north distance error.

Using the AUKF integrated navigation algorithm to combine IMU and GPS data, Figure 4 illustrates the trajectory comparison in 2D between integrated navigation, GPS, IMU, and the true values, along with a detailed local trajectory comparison between 52 m and 60 m eastward. Figure 5 presents the comparison of eastward and northward trajectory errors among integrated navigation, GPS, IMU, and the true values. Table 2 lists the impact of different methods on accuracy. This study uses the Root-Mean-Square Error (RMSE), Position Accuracy Gain (PAG), and Maximum Position Error (MPE) as evaluation metrics.

The RMSE is defined as

(40) $$RMSE=\sqrt{\frac{1}{N}\sum_{i=1}^{N}{\left({\hat{X}}_{i}^{k}-X_{i}^{k}\right)}^{2}}$$

Here, represents the total number of time points, and ${\hat{X}}_{i}^{k}-X_{i}^{k}$ represents the error between the AUKF integrated navigation algorithm and the ground truth at the *i*-th time point.

PAG is used to calculate how much a method’s positioning accuracy improves compared to GPS positioning accuracy [28]. It is calculated as follows:

(41) $$PAG=\frac{RMSE_{GPS}-RMSE_{fusionmethod}}{RMSE_{GPS}}\times 100$$

where $RMSE_{fusionmethod}$ represents the RMSE after applying the fusion method, and $RMSE_{GPS}$ represents the RMSE when using only GPS.

The MPE is used to describe the maximum positioning error compared to the ground truth under the worst conditions.

The data in Table 2 show that the single IMU has the lowest accuracy, while the GPS accuracy is close to the true value. The proposed AUKF integrated navigation algorithm has better accuracy in 2D compared to a single GPS and IMU, with smaller errors in both the east and north directions. Additionally, compared to GPS positioning, the AUKF achieved higher accuracy gains in both the east and north directions, and its MPE in these directions was slightly improved over GPS. This indicates that integrated navigation can achieve more precise trajectory positioning.

**Figure 6:**
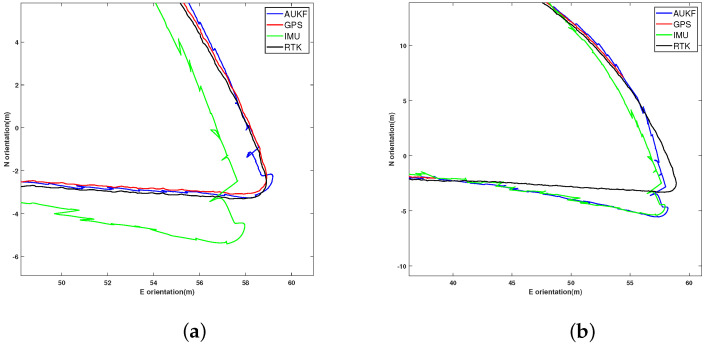
Comparison of 2D planar trajectories among AUKF integrated navigation, GPS, IMU, and ground truth for long distances: (**a**) full GPS signal; (**b**) partial GPS signal failure.

Figure 6 shows the detailed local results of the 2D-plane positioning test, where the GPS signal was artificially blocked for 40 s during a turn to simulate GPS failure. When the GPS signal failed, the integrated navigation system relied on the IMU for trajectory estimation and achieved accurate trajectory recovery after the GPS signal was restored. This validates the ability of the integrated navigation system to maintain trajectory estimation using the IMU during GPS signal loss and accurately correct the trajectory once the signal is restored.

### 4.2. Three-Dimensional Spatial Positioning Verification

**Figure 7:**
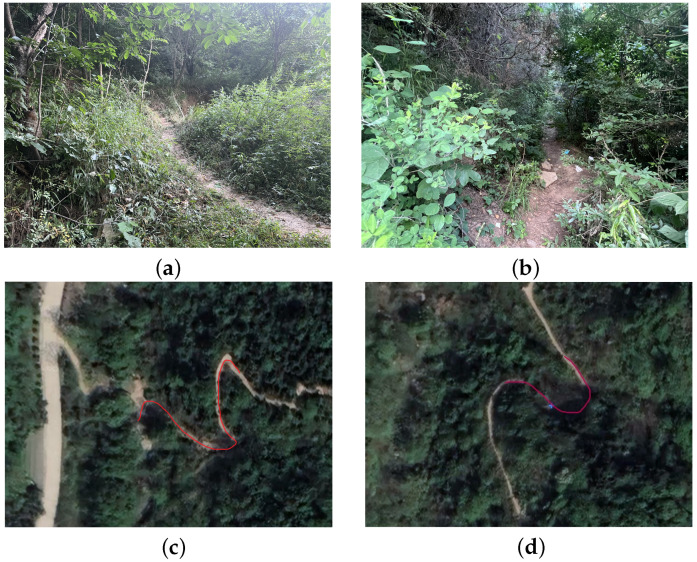
Data collection by rescue personnel walking on rough terrain and roads with GPS signal interruptions: (**a**) rugged terrain, actual view; (**b**) road with GPS signal interruptions, actual view; (**c**) rugged terrain, satellite view; (**d**) road with partial GPS signal interruptions, satellite view.

In the three-dimensional spatial positioning tests within a forest environment, this study further validated the performance of the navigation system on rugged roads and in areas with GPS signal interruptions. The test was conducted on a specific section of road in Ziwuyu, Xi’an, Shaanxi Province, China. Detailed information about the test section is provided in Table 3. This selected route was used to evaluate the navigation capabilities of the system. The road scenario is illustrated in Figure 7.

#### 4.2.1. Rugged Terrain Validation in 3D Space

**Figure 8:**
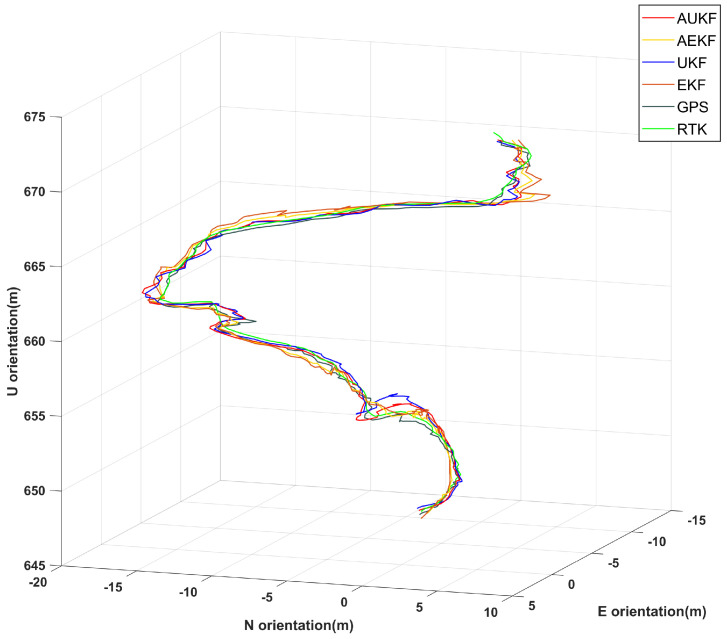
Comparison of 3D trajectories using AUKF, AEKF, UKF, EKF, and RTK on rugged terrain.

**Figure 9:**
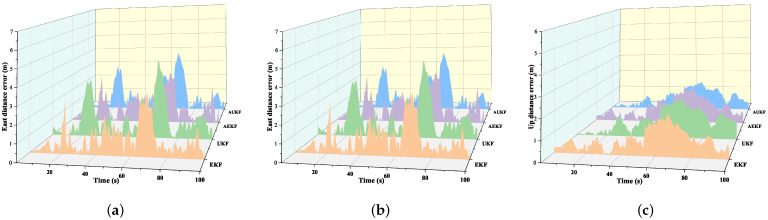
Distance errors on rugged terrain: (**a**) eastward position error; (**b**) northward position error; (**c**) upward position error.

In Figure 8, the navigation paths on rugged roads are represented by different colored lines: the red line indicates the proposed Adaptive Unscented Kalman Filter algorithm, the yellow line represents the AEKF algorithm, and the blue and orange lines correspond to the UKF and EKF algorithms, respectively. The brown and green lines depict the GPS trajectory and the true path obtained via RTK, respectively. The figure demonstrates that, starting from an altitude of 650 m, the navigation paths derived from the EKF and UKF algorithms exhibit significant deviations from the true trajectory, particularly in the altitude and attitude parameters. In contrast, although the AEKF algorithm shows some improvement over the EKF algorithm, the proposed AUKF algorithm more closely follows the true trajectory, with significantly less deviation than the traditional EKF, UKF, and AEKF algorithms. The specific errors are detailed in Figure 9 and Table 4. A comparison of positional errors in three directions reveals that the traditional EKF algorithm has notably higher errors, while the AUKF algorithm demonstrates a clear advantage in this regard.

According to the data presented in Table 4, the AUKF-based fusion positioning method demonstrates significantly higher accuracy compared to the other three methods. This algorithm effectively reduces the RMSE in all directions, achieving an 18.32% reduction in the N direction and an 8.51% reduction in the U direction compared to the EKF. Although the reduction in the E direction is smaller, it still reaches 3.85%. Compared to the adaptive EKF algorithm, the AUKF reduces the error by 11.53% in the N direction and by 6.38% and 5.81% in the E and U directions, respectively. This indicates that adaptive filtering methods can effectively reduce positioning errors in all three directions, with the AUKF showing a more pronounced improvement in positioning accuracy. The primary advantage of the AUKF lies in its ability to adaptively adjust the measurement noise covariance matrix during the filtering process, which allows for the more effective integration of IMU and GPS data, thereby enhancing the overall positioning accuracy.

The proposed AUKF algorithm effectively mitigates the impact of variations in measurement noise. The UKF avoids the divergence risk associated with linearization errors inherent in the EKF by eliminating the linearization process, thereby generally achieving higher estimation accuracy in nonlinear systems. However, since the UKF algorithm does not perform an online estimation of measurement noise characteristics, its accuracy is susceptible to variations in rescue workers’ walking postures on rough terrain, as evidenced by the prominent northward position error shown in Figure 9. Applying an adaptive adjustment of the measurement noise covariance matrix to the EKF algorithm improves system accuracy, indicating that adaptive filtering significantly enhances estimation properties and suppresses outliers in Kalman filtering. Compared to the AEKF, the proposed AUKF algorithm exhibits superior accuracy. Consequently, the AUKF algorithm achieves lower positional errors than the traditional UKF, EKF, and AEKF algorithms in most scenarios involving rugged terrain.

In summary, the proposed AUKF algorithm, by estimating measurement noise parameters in real time, effectively addresses the impact of non-Gaussian noise during state estimation, achieving higher accuracy than the traditional UKF, EKF, and AEKF algorithms. Although the AUKF algorithm increases computational complexity compared to the traditional UKF, the significant improvement in accuracy justifies this trade-off.

#### 4.2.2. Three-Dimensional Space GPS Signal Interruptions Terrain Validation

**Figure 10:**
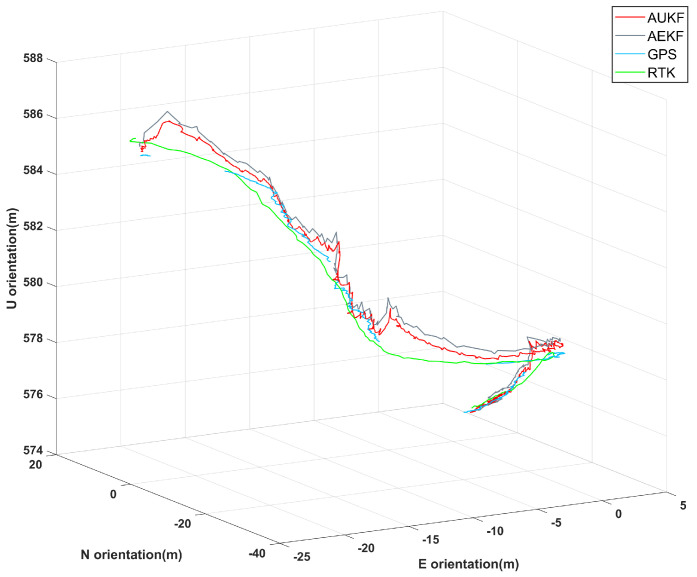
Comparison of 3D trajectories using AUKF, AEKF, GPS, and RTK with partial GPS coverage.

Figure 10 illustrates the performance of the proposed AUKF integrated navigation algorithm in scenarios where GPS signals are interrupted. The red trajectory represents the results from the AUKF algorithm, the gray trajectory corresponds to the AEKF algorithm, the blue trajectory shows the GPS data, and the green trajectory depicts the true path obtained via RTK. Throughout the testing process, four segments lacked GPS signal coverage, with the longest gap lasting 18 s. In areas with GPS signal coverage, the AUKF algorithm’s trajectory closely aligns with both the GPS and true paths, demonstrating superior navigation accuracy. In regions where GPS signals were interrupted, the AUKF algorithm maintained higher continuity and stability compared to the AEKF algorithm.

**Figure 11:**
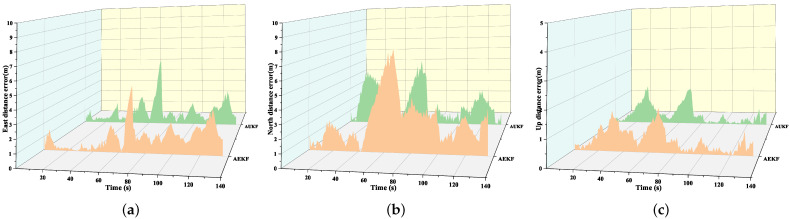
Distance errors with AUKF and AEKF during GPS outages: (**a**) eastward position error; (**b**) northward position error; (**c**) upward position error.

Figure 11 shows the distance errors in the north, east, and vertical directions when there are GPS signal interruptions for both the AUKF and AEKF integrated navigation algorithms. Overall, the largest error occurs in the north direction, followed by the east, with the smallest error in the vertical direction. This result indicates that, during GPS outages, the northward positioning accuracy deteriorates the most significantly, possibly due to environmental factors and walking posture. In comparison, the AUKF algorithm outperforms the AEKF algorithm in all three directions, further highlighting the advantages of the AUKF in complex navigation environments.

**Figure 12:**
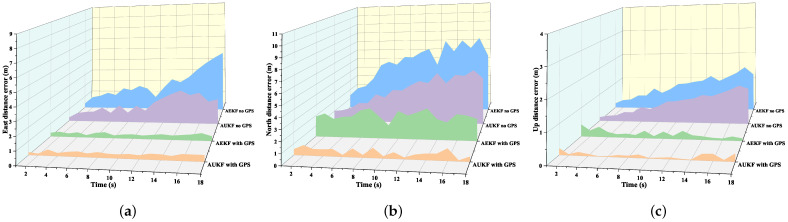
Distance errors using AUKF and AEKF algorithms with and without GPS coverage: (**a**) Eastward position error; (**b**) Northward position error; (**c**) Upward position error.

To evaluate the performance of the AUKF integrated navigation algorithm during GPS interruptions, data from an 18-s GPS outage and an 18-s GPS coverage period were selected for analysis. Figure 12 compares the distance errors in the north, east, and vertical directions for both the AUKF and AEKF algorithms under these two conditions. During the GPS outage, the AUKF algorithm quickly increases the magnitude of the elements in the adaptive covariance matrix, reduces the weighting of GPS measurements, and increases reliance on the rescue worker’s posture model. This strategy allows the AUKF algorithm to rely less on GPS data and more on IMU sensors during GPS outages, resulting in more accurate position estimates that are closer to the true values.

Table 5 compares the RMSEs and MPEs of the proposed AUKF algorithm during GPS outages and coverage in road tests without GPS signal coverage. Table 6 presents the corresponding performance of the AEKF algorithm in these tests. The entire test segment was analyzed in three scenarios for detailed error analysis. When GPS signals were continuously available, the AUKF method showed minimal errors. During GPS outages, the AUKF combined navigation algorithm maintained a certain level of accuracy, but errors significantly increased compared to GPS coverage, especially in the north direction. This is because, without GPS signal coverage, the navigation system relies solely on IMU sensor data, leading to inevitable cumulative errors. However, the AUKF algorithm’s use of an adaptive measurement noise covariance matrix in the corresponding segments significantly reduced the maximum errors in most segments, demonstrating good robustness and adaptability.

## 5. Conclusions

To accurately estimate the positions of rescue personnel in forest environments, this paper proposes an AUKF algorithm that integrates GPS and IMU data, along with barometric altitude measurements, to achieve high-precision 3D positioning in complex environments. Addressing the issue of the decreased filtering accuracy of the UKF under non-Gaussian noise conditions, the proposed AUKF algorithm adaptively adjusts the measurement noise covariance matrix. Specifically, when the GPS measurement accuracy significantly drops, it rapidly increases the amplitude of each element in the covariance matrix, thereby reducing the weight of GPS observations. Additionally, the AUKF algorithm linearizes the nonlinear model and computes the adaptive covariance matrix in real time. It provides precise position estimates of rescue personnel by considering the nonlinearities of both the rescue system and the GPS measurement model. The test results demonstrate that this method not only achieves higher 3D spatial positioning accuracy in forests compared to the traditional EKF, UKF, and adaptive EKF but also enhances the system’s robustness and reliability in complex environments.

## Figures and Tables

**Table 1:** Main specifications of each sensor.

Sensor		Sampling Rate	Measurement Accuracy (Output Noise)	Measurement Range
IMU sensors	Accelerometer (JY901B)	200 Hz	0.75 1 mg—RMS	±16 g
	Gyroscope (JY901B)		0.028∼0.07 (deg/s)—RMS	±2000 deg/s
	Barometer (JY901B)		0.5 Pa—RMS	300∼1100 hPa
GPS sensors	M10050	10 Hz	position: 2.0 m—CEP	
			velocity: 0.05 m/s—RMS	
			course angle: 0.3 deg—RMS	
RTK	QianXunSRmini	1 Hz	horizontal: ±($2.5+0.5\times 10^{-6}$D) mm	
			vertical: ±($5+0.5\times 10^{-6}$D) mm	

**Table 2:** Evaluation metrics for different methods.

Method	Orientation	RMSE (m)	PAG (%)	MPE (m)
IMU	E	1.5936	−149.98	5.048
	N	4.3166	−649.28	12.0848
GPS	E	0.6375	0	2.3728
	N	0.5761	0	1.9273
AUKF	E	0.3014	52.72	1.0655
	N	0.2923	49.26	0.9877

**Table 3:** The coordinates corresponding to different test scenarios.

Test Scenario	Start Longitude	Start Latitude	End Longitude	End Latitude	Altitude Difference (m)
Rugged terrain	108.88437	34.02897	108.88408	34.02888	22.152
Terrain with GPS interruption	108.88429	34.01796	108.88414	34.01787	10.732

**Table 4:** Evaluation metrics for different methods.

Method	Orientation	RMSE (m)	PAG (%)	MPE (m)
EKF	E	1.1421	23.72	3.3767
	N	2.7159	−5.83	8.1469
	U	1.0339	−1.95	1.9770
UKF	E	1.3325	11.00	4.4739
	N	2.4705	24.53	6.6933
	U	0.9751	3.87	1.9489
AEKF	E	1.1729	21.69	3.2335
	N	2.5075	2.34	7.6206
	U	1.0042	1.01	1.7159
AUKF	E	1.0981	26.66	3.8814
	N	2.2184	13.55	5.8957
	U	0.9459	6.75	1.5588

**Table 5:** Evaluation metrics for different states in AUKF algorithm.

State of Operation	Time (s)	Orientation	RMSE (m)	MPE (m)
Complete process	140	E	1.0636	2.6108
		N	2.3744	5.1931
		U	0.5102	1.3444
No GPS coverage	18	E	1.5907	2.6108
		N	3.5057	5.1931
		U	0.8014	1.3444
With GPS coverage	18	E	0.3473	0.4631
		N	0.5731	1.0230
		U	0.2159	0.2441

**Table 6:** Evaluation metrics for different states in AEKF algorithm.

State of Operation	Time (s)	Orientation	RMSE (m)	MPE (m)
Complete process	140	E	1.2645	4.8705
		N	2.7402	7.5571
		U	0.5778	1.6192
No GPS coverage	18	E	1.7429	4.8705
		N	3.9408	7.5571
		U	0.9421	1.6192
With GPS coverage	18	E	0.4169	0.5497
		N	0.6905	2.8092
		U	0.2621	0.4135

## Data Availability

The data supporting the reported results in this study are not publicly available due to privacy restrictions.
